# Association between suicidal ideation and cognitive function in young patients with schizophrenia spectrum disorder

**DOI:** 10.3389/fpsyt.2023.1276511

**Published:** 2023-10-27

**Authors:** Ji-Su Kim, Seon-Hwa Baek, Honey Kim, Ju-Wan Kim, Hee-Ju Kang, Seunghyong Ryu, Ju-Yeon Lee, Jae-Min Kim, Sung-Wan Kim

**Affiliations:** ^1^Department of Psychiatry, Chonnam National University Medical School, Gwangju, Republic of Korea; ^2^Mindlink, Gwangju Bukgu Mental Health Center, Gwangju, Republic of Korea

**Keywords:** suicide, cognitive function, schizophrenia, early psychosis, depression

## Abstract

**Introduction:**

Suicide is a major concern for patients with recent-onset schizophrenia. We hypothesized that preserved cognitive function might be associated with a higher level of suicidality in patients with schizophrenia. We investigated the associations between cognitive function and suicidal ideation (SI) in young patients recently diagnosed with a psychotic disorder.

**Methods:**

This study analyzed data from a naturalistic clinical cohort study that comprised 402 patients with schizophrenia spectrum disorder. Patients with a treatment duration of ≤5 years and an age range of 15–39 years were enrolled. Participants were categorized into two groups based on SI as assessed by the Columbia Suicidal Severity Rating Scale. We collected demographic and clinical data and administered psychiatric, neurocognitive, and social cognitive measures.

**Results:**

Among participants, 52% reported experiencing SI. Patients with SI were significantly younger and had a longer duration of untreated psychosis (DUP) than those without it. The Positive and Negative Syndrome Scale-general psychopathology score was significantly higher in the SI group. Scores on the Calgary Depression Scale for Schizophrenia, Perceived Stress Scale, Beck Depression Inventory (BDI), and Beck Hopelessness Scale were significantly higher among patients with SI, while scores on the Subjective Well-being Under Neuroleptics-Short Form and Brief Resilience Scale were significantly lower compared to those without it. Patients with SI demonstrated significantly higher scores on the verbal and visual learning test, false belief task, picture stories task, and Controlled Oral Word Association Test. They also completed the Trail Making Test (TMT) parts A and B in significantly less time than those without it. After adjusting for age, DUP, and scores on the BDI, group differences in scores on the verbal and visual learning tests, TMT (parts A and B), and the false belief task, and the picture story task remained significant.

**Discussion:**

Our results suggest that along with traditional risk factors, better cognitive function may also be a major risk factor for suicidality in patients with schizophrenia. Providing psychological support and cognitive interventions is essential for young patients with recent-onset schizophrenia spectrum disorders, particularly those with high levels of depression, hopelessness, perceived stress, low resilience, and good cognitive function.

## Introduction

Suicide ranks as the fifth leading cause of death in South Korea (1) and imposes a significant socioeconomic burden (2). In individuals with schizophrenia, suicide is a tragic outcome, accounting for early mortality in nearly 5% of patients, with 25–50% attempting suicide during their lifetime (3, 4). Despite the gravity of this issue, the relatively low incidence of completed suicide in this population makes comprehensive investigation challenging (5). Suicidal behaviors, encompassing both suicide attempts and suicidal ideation (SI), are prevalent among individuals with schizophrenia and are recognized as substantial risk factors for suicide (6, 7). Hence, the identification of factors associated with suicidal behavior in patients with schizophrenia plays a crucial role in suicide prevention efforts. Previous research has revealed that factors such as previous suicide attempts, the choice of more lethal methods, feelings of hopelessness, and the presence of depressive symptoms significantly contribute to suicide risk in individuals with schizophrenia, and these results are similar to those found in the general population (8–14).

Cognitive function is one of the domains predominantly affected in patients with schizophrenia spectrum disorder (14). Cognitive functions, such as attention, executive function, working memory, and episodic memory, are impaired in patients with schizophrenia (15). Previous studies have reported that preserved cognitive function in schizophrenia is a risk factor for suicidality (16, 17). However, these studies were conducted in patients with chronic schizophrenia. Young patients with schizophrenia face a significantly higher risk of suicidality (18–23). Additionally, the highest risk of suicide occurs within the first 5 years after diagnosis (4, 24). Therefore, it is crucial to identify the risk factors for suicidality in young patients with recently diagnosed schizophrenia.

In young patients with psychotic disorders, suicidal thoughts are strongly linked to actual suicidal behavior and even death by suicide (20–22). When patients experience SI, they may be at an elevated risk of taking further steps toward suicide. Furthermore, SI is a crucial element in the overall understanding of how individuals progress from having thoughts of suicide to engaging in suicidal behaviors. Thus, for effective suicide prevention in patients with early psychosis, it is necessary to investigate the pathogenesis of SI.

We hypothesized that preserved cognitive function might be associated with a higher level of suicidality in patients recently diagnosed with schizophrenia. Most previous studies examining the relationship between neurocognitive function and suicidality were conducted in patients with chronic schizophrenia and did not incorporate measures assessing social cognition and theory of mind. Therefore, in this study, we investigated the associations between cognitive function, including social cognition, and SI in young patients recently diagnosed with a schizophrenia spectrum disorder.

## Materials and methods

### Study design

We analyzed the data from a naturalistic clinical cohort study conducted at the Gwangju Early Treatment and Intervention Team of Chonnam National University Hospital (25). All participants were consecutively recruited from patients who recently developed psychotic symptoms and visited Chonnam National University Hospital. In this study, inclusion criteria were a treatment duration of ≤5 years and an age range of 15–39 years. Furthermore, a qualified research psychiatrist established the clinical diagnosis for each participant, ensuring that all patients met the criteria for ‘Schizophrenia Spectrum Disorder and Other Psychotic Disorders’ in the Diagnostic and Statistical Manual of Mental Disorders, Fifth Edition (26). Patients with a substance-or medication-induced psychotic disorder, psychotic disorder due to another medical condition, or severe neurological or medical disorder were excluded. The study was conducted from September 2015 to December 2022, during which a total of 402 participants met the inclusion criteria. This study was approved by the Chonnam National University Hospital Institutional Review Board, and all subjects provided written informed consent before participating.

### Demographic and clinical measures

Baseline sociodemographic and clinical data were collected, including age, sex, education level, occupation, body mass index (BMI), duration of treatment, diagnosis, and duration of untreated psychosis (DUP; time between the appearance of the initial psychotic symptoms and the start of antipsychotic treatment or psychiatric hospitalization) (27). The doses of prescribed antipsychotics were converted into chlorpromazine (CPZ)-equivalent dose (28).

Suicidal behavior was assessed using a semi-structured interview with the Columbia Suicidal Severity Rating Scale (C-SSRS) administered by a trained researcher (29, 30). At the baseline assessment, the C-SSRS was used to evaluate lifetime suicidal behavior. Patients who responded affirmatively to any item indicative of suicidal ideation (SI) were categorized into the SI group. Psychiatric symptoms were evaluated using the Positive and Negative Syndrome Scale (PANSS) (31, 32). We utilized three subscales (positive, negative, and general psychopathology) as well as total scores as variables. For exploratory analysis, we also examined the 12th item of the general psychopathology subscale (lack of insight and judgment; G12). Other objective psychiatric measures included Social and Occupational Functioning Assessment Scale (SOFAS; used to evaluate general functioning) (33) and Calgary Depression Scale for Schizophrenia (CDSS; used to analyze depressive symptoms) (34, 35).

We employed several subjective self-report measures, including the Beck Depression Inventory (BDI; used to assess depressive symptoms) (36, 37), Beck Hopelessness Scale (BHS; used to evaluate hopelessness) (38, 39), Perceived Stress Scale (PSS; used to assess subjective stress) (40, 41), Brief Resilience Scale (BRS; used to quantify the ability to recover from stress) (42), and Subjective Well-being Under Neuroleptics-Short Form (SWN-K; used to evaluate subjective health-related quality of life) (43). Higher scores on the BRS and SWN-K, and lower scores on the BHS, BDI, and PSS, indicate better outcomes.

### Neurocognitive measures

We assessed neurocognitive function using a computerized neurocognitive test battery standardized for the Korean population, including individuals with psychotic disorders. Research nurses, who were unaware of the study’s objectives, administered six neurocognitive tests. The Digit Span test was used to assess attention span; higher scores indicate superior attention and vigilance (44–46). The modified Rey Auditory Verbal Learning Test was used to assess immediate and delayed verbal memory; the outcome measure is the total number of recalls (46, 47). The visual learning test was administered to assess immediate and delayed visual memory. The outcome measure was the total number of recalls, and a higher score indicated better memory (48). The Wisconsin Card Sorting Test was administered to assess executive function; the outcome measure is the number of categories completed, and a higher score indicates better executive function and cognitive flexibility (49–51). The Continuous Performance Test (CPT) was administered to measure the ability to sustain attention (vigilance) to a stimulus; the outcome measures are reaction time and number of correct responses, where faster reaction times and more correct responses indicate superior attention (52–54). Finally, the Trail Making Test (TMT) was used to assess visuomotor coordination. The TMT consists of two parts that measure executive function and visuospatial working memory (A and B, respectively), and the outcome measure is the time taken (in seconds) to complete both parts (54–56). In addition, we used the Mini-Mental Status Examination (MMSE) to assess global cognitive function (57–59) and the Controlled Oral Word Association Test (COWAT) to evaluate verbal fluency and cognitive speed (60, 61).

### Social cognitive measures

The false belief task of Wimmer and Perner was administered to assess social cognition (62, 63). Patients are required to infer the thoughts of characters in four stories. The maximum total score is 12 points, and higher scores indicate the superior theory of mind (ToM) and social cognition. Picture stories task developed by Brüne were used to assess ToM, which is the ability to infer others’ mental states and emotions (64, 65). Six cartoons are presented to the patient without a background story description, and the patient is then required to answer questions related to ordering and mind-reading. This task represents a more complex and higher-order ToM test. Total ordering (36 points) and mind-reading (23 points) scores are calculated; higher scores indicate superior ToM (65).

### Statistical analysis

The dependent variable in this study was lifetime suicidal ideation, assessed using the C-SSRS. Participants were divided into two groups based on the presence of SI. Sociodemographic and clinical characteristics, as well as cognitive function, were compared between these two groups. We used the chi-square test for categorical variables, the independent t-test for normally distributed variables, and the Mann–Whitney *U* test for variables that did not follow a normal distribution, as appropriate. To control for the potential confounding effects of age, DUP, and BDI scores, which showed significant associations with SI in the univariate analyses, we included them as covariates in logistic regression analysis along with each cognitive measure that demonstrated a significant association with SI in the univariate analyses. DUP and BDI scores, which were not normally distributed, were log-transformed before entering them into the model. TMT part A and B times, also not normally distributed, were transformed into 10-point scales based on ten percentiles before inclusion in the logistic regression analysis. For exploratory analysis, we calculated Pearson’s or Spearman’s correlation coefficients between the PANSS G12 score and measures of clinical status and ToM. All statistical tests were two-tailed, and *p* < 0.05 was taken to indicate statistical significance. The statistical analysis was performed using SPSS software (version 25.0; IBM Corp., Armonk, NY, United States).

## Results

In total, 402 patients (males, 49.2%; females, 50.2%) with recent-onset psychosis were included in the analysis. The mean (standard deviation) age at baseline was 24.4 (5.7) years. The most common diagnosis was schizophrenia (*n* = 244, 65.9%), followed by schizophreniform disorder (*n* = 78, 21.1%), other specified schizophrenia spectrum disorder (*n* = 34, 9.2%), and schizoaffective disorder (*n* = 14, 3.8%). Approximately half of the patients (52.0%) reported experiencing SI.

Table 1 shows the participants’ sociodemographic and clinical characteristics according to the presence of SI. Patients with SI were significantly younger and had longer DUP than those without it. There was no significant group difference in sex, marital status, education level, occupation, BMI, duration of treatment, diagnosis, or CPZ equivalent dose.

**Table 1:** Comparison of the sociodemographic and clinical characteristics of participants according to the presence of suicide idea.

	Total (N = 402)	Suicide idea (+) (*N* = 209, 52.0%)	Suicide idea (−) (*N* = 193, 48.0%)	Statistical value	*p*-value
Age, mean (SD) years	24.4 (5.7)	23.5 (5.2)	25.4 (6.1)	*T* = −3.229	0.001
Gender, *N* (%) women	202 (50.2)	107 (51.2)	95 (49.2)	*χ^2^ =* 0.156	0.693
Marital status, *N* (%) married	22 (5.5)	7 (3.3)	15 (7.8)	*χ^2^* = 3.794	0.051
Education, median (IQR) year	14.0 (12.0–16.0)	13 (12–16)	14 (12–16)	*Z* = −1.331	0.183
Occupation, *N* (%) Employed	95 (23.6)	51 (24.4)	44 (22.8)	*χ^2^* = 0.143	0.705
Duration of treatment, median (IQR) month	1.0 (0.8–3.2)	1.0 (0.9–3.2)	1.0 (0.8–3.5)	*Z* = −0.229	0.819
DUP, median (IQR) month	3.2 (1.0–15.0)	6.0 (1.0–18.0)	2.0 (1.0–8.75)	*Z* = −2.979	0.003
Diagnosis, *N* (%) Schizophrenia	244 (65.9)	116 (61.7)	128 (70.3)	*χ*^2^ = 6.596	0.086
Schizophreniform	78 (21.1)	40 (21.3)	38 (20.9)		
Schizoaffective disorder	14 (3.8)	8 (4.3)	6 (3.3)		
Other specified SSD	34 (9.2)	24 (12.8)	10 (5.5)		
CPZ equivalent dosage, median (IQR) mg/day	400 (200–600)	325 (200–600)	400 (200–650)	*Z* = −1.580	0.114
Body Mass Index, median (IQR)	22.2 (20.0–24.9)	21.9 (20.0–25.2)	22.2 (20.6–24.7)	*Z* = −0.094	0.925
PANSS, Positive, mean (SD)	15.1 (4.8)	15.4 (4.8)	14.7 (4.8)	*T* = 1.294	0.197
Negative, mean (SD)	16.2 (4.7)	16.0 (4.6)	16.4 (4.8)	*T* = −0.836	0.404
General psychopathology, mean (SD)	33.8 (7.5)	34.9 (7.5)	32.6 (7.4)	*T* = 3.152	0.002
Total, mean (SD)	65.0 (14.6)	66.1 (14.6)	63.8 (14.6)	*T* = 1.540	0.124
G12 (lack of insight), mean (SD)	3.1 (1.2)	3.1 (1.1)	3.2 (1.2)	*T* = −1.267	0.206
SOFAS, mean (SD)	60.3 (9.9)	60.7 (10.2)	59.8 (9.6)	*T* = 0.913	0.362
CDSS, median (IQR)	4.0 (10–7.0)	5.0 (2.0–9.0)	2.0 (0.75–5.0)	*Z* = −6.810	<0.001
SWN-K, mean (SD)	75.5 (17.4)	70.4 (17.4)	81.3 (15.6)	*T* = −6.147	<0.001
Brief Resilience Scale, mean (SD)	17.1 (4.9)	16.1 (4.9)	18.3 (4.5)	*T* = −4.445	<0.001
Perceived Stress Scale, mean (SD)	20.3 (6.5)	21.9 (6.3)	18.5 (6.2)	*T* = 5.015	<0.001
Beck Depression Inventory, median (IQR)	7.0 (3.0–16.8)	12.0 (5.0–23.0)	5.0 (1.0–10.0)	*Z* = −6.822	<0.001
Beck Hopelessness Scale, mean (SD)	3.1 (1.0)	3.3 (1.0)	2.9 (1.0)	*T* = 3.200	0.001

SD, standard deviation; IQR, Inter-Quartile Range; DUP, Duration of Untreated Psychosis; SSD, Schizophrenia Spectrum Disorder; PANSS, CPZ, chlorpromazine; Positive and Negative Syndrome Scale; SOFAS, Social and Occupational Functioning Assessment Scale; CDSS, Calgary Depression Scale for Schizophrenia; SWN-K, Subjective Well-being Under Neuroleptics-Short Form. All *p*-values are empirical.

The PANSS positive, negative, G12, and total scores did not differ according to SI, but the general psychopathology score was significantly higher in the SI group. Scores on the CDSS, PSS, BDI, and BHS were significantly higher in patients with SI compared to those without it, whereas scores on the SWN-K and BRS were significantly lower. There were no significant associations found between G12 and other clinical measures (data not shown). However, the PANSS G12 score was significantly correlated with scores on the picture stories task and the false belief task (*r* = −0.130, *p* = 0.009, and *r* = −0.117, *p* = 0.019, respectively).

Table 2 shows the neurocognitive and social-cognitive outcomes according to the presence of SI. The patients with SI had significantly higher scores on the verbal and visual learning test, false belief task, picture stories task, and COWATT, and took significantly less time to complete TMT parts A and B, than those without it. They also completed more categories, although the difference was not statistically significant (*p* = 0.084). The two groups had no significant differences in scores on the digit span test, CPT, or MMSE.

**Table 2:** Comparison of neurocognitive and social cognitive outcomes of participants according to the presence of suicide idea.

	Suicide idea (+) (*N* = 209, 52.0%)	Suicide idea (−) (*N* = 193, 48.0%)	Statistical value	*p-*value
Digit Span, Forward, mean (SD)	7.2 (1.5)	7.1 (1.4)	*T* = 0.554	0.580
Digit Span, Backward, mean (SD)	5.5 (1.7)	5.3 (1.4)	*T* = 1.447	0.149
Verbal Learning Test, total mean (SD)	89.8 (18.5)	85.9 (20.1)	*T* = 2.018	0.044
Visual Learning Test, total mean (SD)	73.3 (12.1)	69.6 (13.8)	*T* = 2.773	0.006
WCST, Categories completed, median (IQR)	6.0 (4.0–6.0)	6.0 (4.0–6.0)	*Z* = −1.725	0.084
CPT, Correct Response, mean (SD)	116.6 (21.2)	115.7 (20.2)	*T* = 0.413	0.680
CPT, Reaction Time, mean (SD) millisecond	682.6 (70.5)	686.6 (64.5)	*T* = −0.592	0.554
TMT, Part A, median (IQR) second	22.0 (18.0–27.0)	23.0 (19.0–30.0)	*Z* = −2.089	0.037
TMT, Part B, median (IQR) second	38.0 (30.0–49.0)	42.0 (34.0–58.0)	*Z* = −3.407	0.001
False Belief Task. mean (SD)	8.3 (2.7)	7.6 (2.3)	*T* = 2.728	0.007
Picture Stories Task. mean (SD)	50.3 (8.4)	48.1 (9.1)	*T* = 2.572	0.010
Controlled Oral Word Association Test, mean (SD)	69.4 (20.7)	65.1 (17.9)	*T* = 2.226	0.027
Mini-Mental Status Examination, mean (SD)	28.5 (1.6)	28.5 (1.6)	*T* = −0.157	0.875

SD, standard deviation; IQR, Inter-Quartile Range; WCST, Wisconsin Card Sorting Test; CPT, Continuous Performance Test; TMT, Trail Making Test.

Table 3 shows the results of adjusted logistic regression analyses. After controlling for age, DUP, and scores on the BDI, significant differences between groups remained evident in scores on the verbal and visual learning tests, TMT (parts A and B), the false belief task, and the picture story task. However, the group differences in COWATT scores lost statistical significance after adjusting for the confounding variables.

**Table 3:** Adjusted analysis for associations between neurocognitive and social cognitive outcomes and suicidal ideation.

	*B*	Odds ratio (95% confidence interval)	Adjusted *p*-value^*^	Nagelkerke *R^2^*
Verbal Learning Test, 1 score increase	0.012	1.012 (1.000–1.024)	0.047	0.206
Visual Learning Test, 1 score increase	0.028	1.028 (1.010–1.046)	0.002	0.223
TMT, Part A, 10 percentile increase	−0.090	0.914 (0.846–0.987)	0.021	0.200
TMT, Part B, 10 percentile increase	−0.116	0.890 (0.823–0.963)	0.004	0.212
False Belief Task, 1 score increase	0.089	1.093 (1.000–1.194)	0.049	0.193
Picture Stories Task, 1 score increase	0.026	1.026 (1.001–1.052)	0.043	0.194
Controlled Oral Word Association Test, 1 score increase	0.009	1.009 (0.998–1.021)	0.121	0.193

^*^Adjusted for age, duration of untreated psychosis, and the Beck Depression Inventory scores by pairwise logistic regression analysis. All *p*-values are empirical.

## Discussion

This study investigated the risk factors for SI in young patients with recent-onset schizophrenia and examined their association with cognitive function. Patients with SI had superior neurocognitive and social-cognitive function than those without it. In addition, they showed higher levels of depression, perceived stress, and hopelessness and lower levels of resilience and subjective well-being. These results suggest that, along with traditional risk factors such as depression, perceived stress, and hopelessness (8, 9), better cognitive function may also be a major risk factor for suicidality. A better understanding of SI may help prevent suicidal behavior in patients with recent-onset schizophrenia.

The SI group in this study was significantly younger than the group without SI. This finding aligns with the nationwide Danish OPUS trial, which investigated the effectiveness of early intervention programs for schizophrenia spectrum disorders and demonstrated that younger age was a risk factor for suicidality (21). Notably, there is a trend toward a higher risk of suicide at a younger age in patients with schizophrenia compared to the general population, where the risk for suicide tends to be higher among older individuals (23). Individuals diagnosed with schizophrenia often undergo a mourning process as they grapple with the losses experienced upon the onset of their illness (66). The risk of suicide may increase when young individuals with schizophrenia experience demoralization stemming from their pessimistic outlook on a future characterized by long-term treatment demands. Additionally, individuals experiencing their first episode of schizophrenia in life often find themselves in a more unstable situation, as they are unfamiliar with the disorder and, as adolescents, face the typical problems and conflicts that come with beginning a new phase in life (18). Therefore, it is crucial to assess the risk of suicidality when treating young patients with early psychosis, particularly those in their late teens and early twenties.

Longer DUP, a prognostic factor associated with poorer overall outcomes in patients with schizophrenia (67, 68), was observed in the group with SI in this study. SI may occur as a result of protracted psychotic symptoms when the DUP is extended (22). Several studies have demonstrated that a long DUP is a risk factor for suicidality (69, 70), and this association may be mediated by depressive symptoms (71, 72). Furthermore, patients with an extended DUP might experience great distress due to psychotic symptoms, which, in turn, could increase the risk of SI.

As in previous studies of patients with schizophrenia and the general population, the individuals in this study who had experienced SI showed significantly higher levels of depression and hopelessness (11, 73–76). A higher general psychopathology PANSS score in patients with SI might be associated with depressive symptoms. Depression is common in patients with schizophrenia (77), and it should be managed to prevent suicide (78). Hopelessness is a major psychological risk factor and a clinical endophenotype for suicidality in the general population. Hopelessness plays a role in the development of suicidal thoughts and behaviors, and it also mediates the relationship between SI and suicidality (75). Demoralization, which is associated with hopelessness (79), is important in the context of the current study, which explored the link between cognitive function and SI. People who experience hopelessness tend to isolate themselves and not seek help (75). Life stressors can reduce the will to live, and it is intuitive that hopelessness plays an important role in the suicidality of patients with schizophrenia (79).

In recent studies, perceived stress was found to be significantly associated with suicidality in patients with schizophrenia spectrum disorder (80, 81), and resilience is a major protective factor for suicidality. In one study, early psychosis patients with comorbid depression showed lower resilience. Resilience in patients with schizophrenia spectrum disorders was associated with symptom remission, recovery, better social and interpersonal functioning, and higher quality of life, all of which may have reduced SI (82).

In this study, the group with SI demonstrated better performance on several neurocognitive and social-cognitive tasks. Higher levels of verbal and visual learning ability, visuospatial working memory, and ToM were observed in individuals with high suicidality, while attention span, sustained attention, and verbal fluency showed no significant differences between groups. These findings suggest that complex cognitive processes, such as working memory and social cognition, are more strongly associated with suicidality than simple attention.

ToM refers to the ability to understand and represent another person’s mental state and use these representations to explain and predict human interactions (83). It is associated with cognitive flexibility (84, 85), which generally exhibits an inverse association with suicidality (86). However, in this study, the SI group displayed superior ToM even after adjusting for confounding variables. It is possible that patients with high mental reasoning abilities were more aware of the difficulties that can arise during interpersonal and social situations, which could lead to psychological strain (12, 66). This aligns with the significant increase in suicide attempts observed among patients shortly after discharge, when real-world life challenges may escalate (87). Clinical insight, which refers to an individual’s awareness of having a mental illness that requires treatment, can be associated with ToM (88). In this study, ToM was significantly linked to higher levels of insight and judgment, even though there was not difference based on the presence of SI.

Higher levels of insight in patients with schizophrenia can also be connected to increased severity of depression and hopelessness (16, 89). While this study did not demonstrate these associations, it can be inferred that SI may manifest in patients who recognize their own condition, especially those who struggle to maintain social relationships. Providing care for individuals with schizophrenia who are aware of their condition is essential to help them adapt to society, mitigate depressive responses, and reduce the risk of suicide (78). Cognitive therapy and psychological support for patients with early psychosis, particularly those with good cognitive function, are essential to prevent suicidality.

No significant differences in indicators of disease severity, such as the PANSS positive symptoms score, medication dosage, and social functioning, were observed between the groups in this study with and without SI. In previous studies, severe psychotic symptoms such as command hallucinations were associated with suicidality in patients with schizophrenia (23, 90). Relatively low scores on the PANSS among our patients in the early stages of schizophrenia might explain this discrepancy.

This study had some limitations. First, it used a cross-sectional design such that causal relationships could not be established. Therefore, longitudinal studies are needed. Second, a control group of healthy individuals was not included; doing so may have helped us achieve a deeper understanding of the impact of social cognition on suicidality in patients with schizophrenia through comparison with the general population. Third, the generalizability of the results to chronic schizophrenia patients with more severe cognitive impairment may be limited. Fourth, in this study, we used lifetime SI as a key variable for assessing suicidality. Future research should consider incorporating the severity of suicidal behavior. Finally, the potential type I errors should be considered because this study employed empirical value of *p*-values without correction for multiple testing. Nevertheless, our results serve as a basis for future investigations into the relationship between suicidality and cognitive function in young patients with psychosis. We believe that our study contributes to a deeper understanding of suicidal behavior in young patients going through their first episode of schizophrenia and can aid in the development of suicide prevention strategies for this population.

In conclusion, our findings suggest that patients with recent-onset psychosis, who exhibit relatively high cognitive abilities, especially in complex functions and social cognition, are more prone to experiencing suicidal thoughts, potentially leading to increased suicidality. It is crucial to pay greater attention to suicidality among younger patients who demonstrate a better ability to understand others’ perspectives and adapt to social situations. Moreover, providing psychological support with empathy and cognitive interventions is essential for young patients with recent-onset schizophrenia spectrum disorders, particularly those with elevated levels of depression, hopelessness, perceived stress, low resilience, and good cognitive function.

## Data availability statement

The raw data supporting the conclusions of this article will be made available by the authors, without undue reservation.

## Ethics statement

The studies involving humans were approved by Chonnam National University Hospital Institutional Review Board. The studies were conducted in accordance with the local legislation and institutional requirements. The participants provided their written informed consent to participate in this study.

## Author contributions

JSK: Writing – original draft, Data curation, Investigation. SHB: Data curation, Investigation, Methodology, Writing – review & editing. HK: Data curation, Investigation, Writing – review & editing. JWK: Data curation, Investigation, Writing – review & editing. HJK: Writing – review & editing, Conceptualization, Supervision. SR: Supervision, Writing – review & editing, Data curation, Formal analysis. JYL: Supervision, Writing – review & editing. JMK: Supervision, Writing – review & editing. SWK: Writing – review & editing, Conceptualization, Formal analysis, Funding acquisition, Methodology, Writing – original draft.
